# Supplementary material for: DMRforPairs: identifying Differentially Methylated Regions between unique samples using array based methylation profiles
Source: BMC Bioinformatics. 2014 May 15;15:141. doi: 10.1186/1471-2105-15-141 (PMC4046028; doi:10.1186/1471-2105-15-141)
Supplement: Additional file 2 — DMRforPairs output for the comparison of A431-MCF7 and NA17018-NA17105. Please start from the HTML files in each folder. Available via the BMC Bioinformatics website. [file 1471-2105-15-141-S2.zip › 1394847754114233_MOESM2_ESM/A431_MCF7/figures/22368.pdf]

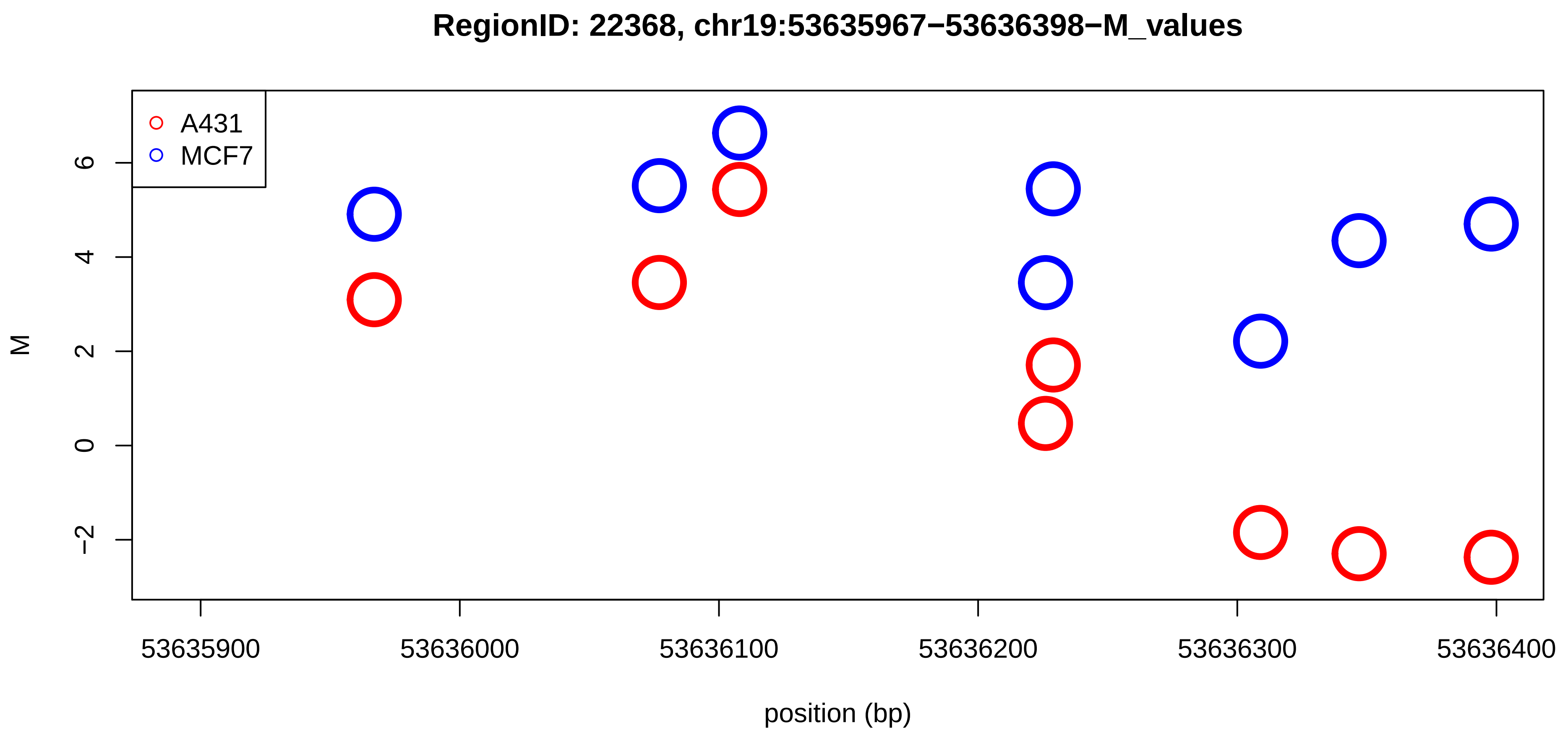

RegionID: 22368, chr19:53635967−53636398−M_values
A431
MCF7
M
−2
0
2
4
6
53635900
53636000
53636100
53636200
53636300
53636400
position (bp)

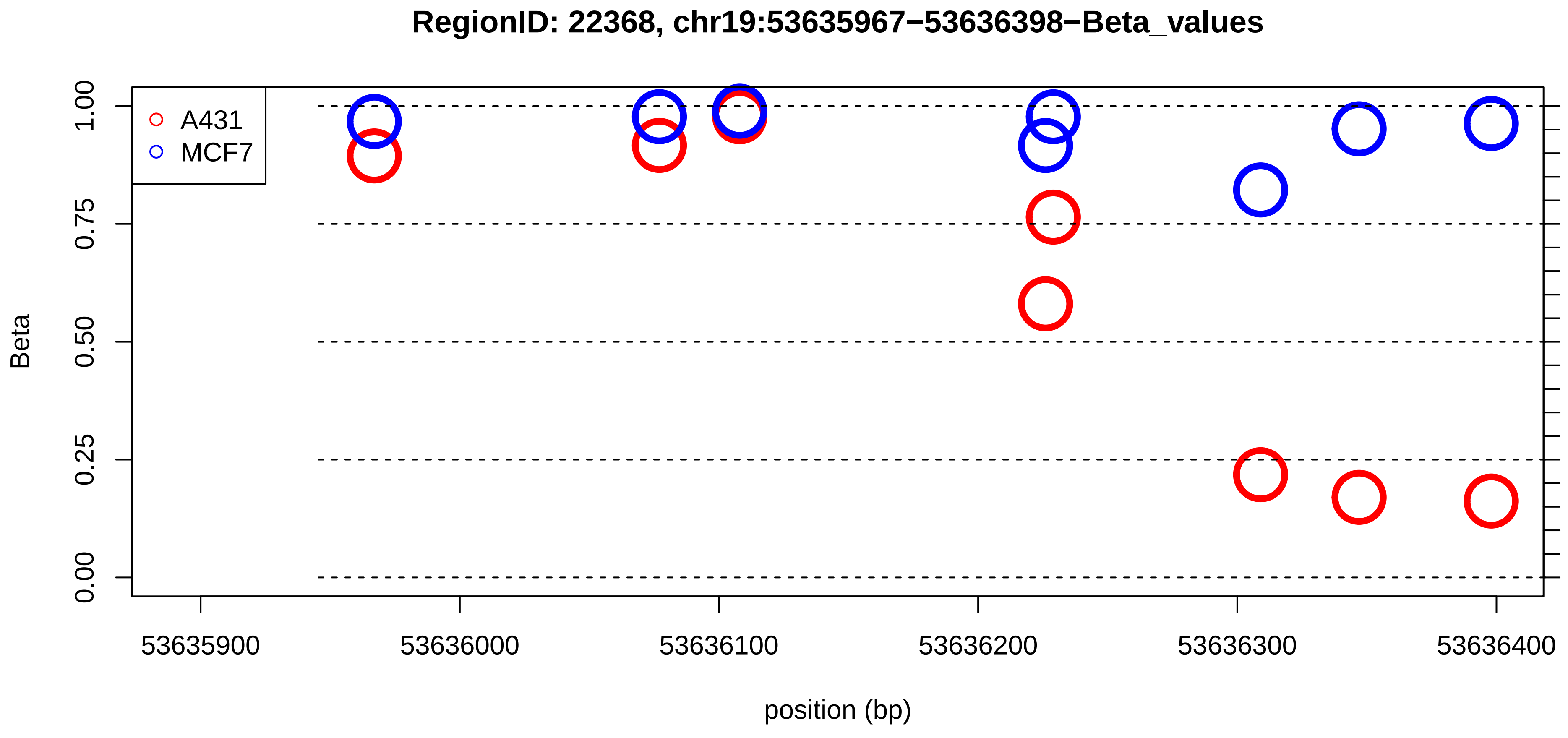

RegionID: 22368, chr19:53635967−53636398−Beta_values
A431
MCF7
Beta
0.00
0.25
0.50
0.75
1.00
53635900
53636000
53636100
53636200
53636300
53636400
position (bp)

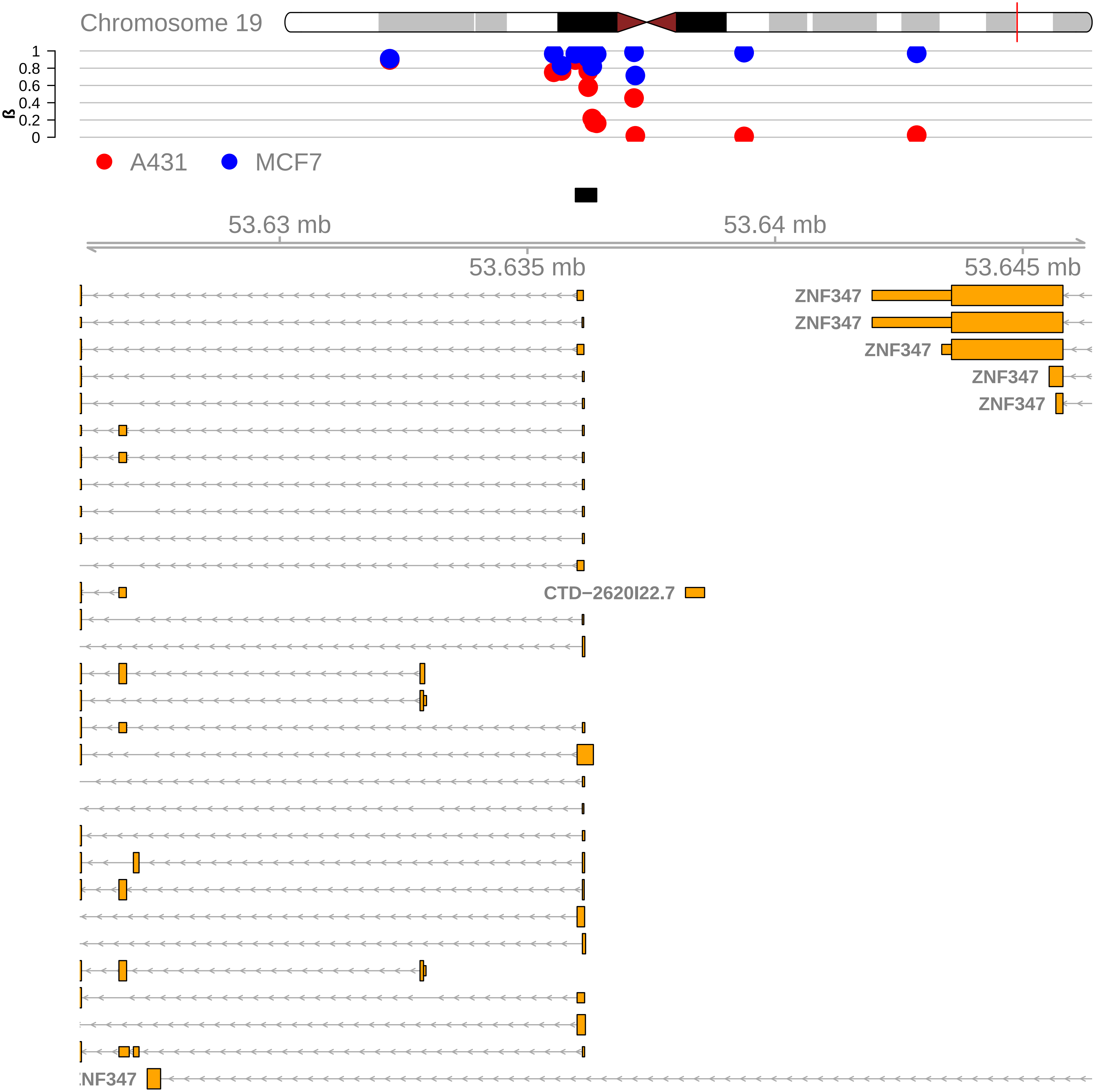

Chromosome 19
ß
1
0.8
0.6
0.4
0.2
0
A431
MCF7
53.63 mb
53.64 mb
53.635 mb
53.645 mb
ZNF347
ZNF347
ZNF347
ZNF347
ZNF347
CTD−2620I22.7
